# Hydrogen Production by Three-Stage (i) Pyrolysis, (ii) Catalytic Steam Reforming, and (iii) Water Gas Shift Processing of Waste Plastic

**DOI:** 10.1021/acs.energyfuels.2c02934

**Published:** 2023-02-14

**Authors:** Rayed Alshareef, Mohamad A. Nahil, Paul T. Williams

**Affiliations:** School of Chemical & Process Engineering, University of Leeds, Leeds LS2 9JT, U.K.

## Abstract

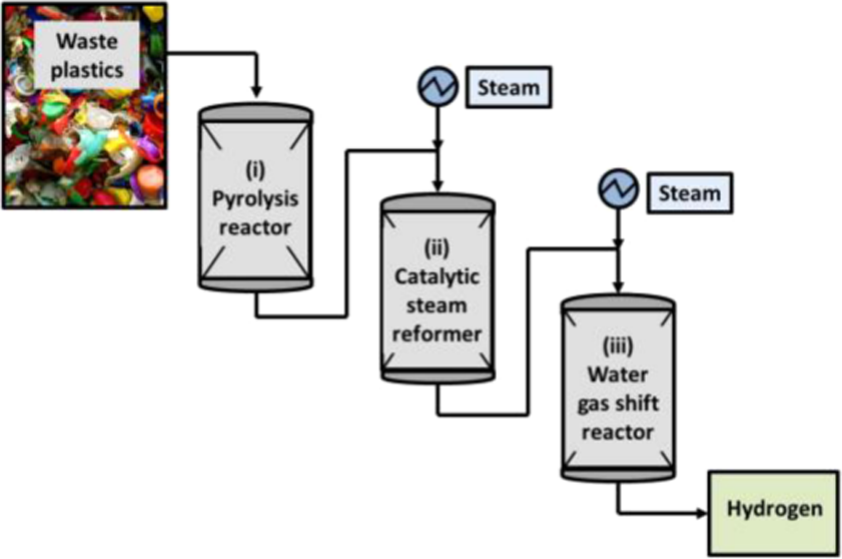

The three-stage (i) pyrolysis, (ii) catalytic steam reforming, and (iii) water gas shift processing of waste plastic for the production of hydrogen have been investigated. The (i) pyrolysis and (ii) catalytic steam reforming process conditions were maintained throughout, and the experimental program investigated the influence of process conditions in the (iii) water gas shift reactor in terms of catalyst type (metal–alumina), catalyst temperature, steam/carbon ratio, and catalyst support material. The metal–alumina catalysts investigated in the (iii) water gas shift stage showed distinct maximization of hydrogen yield, which was dependent on the catalyst type at either higher temperature (550 °C) (Fe/Al_2_O_3_, Zn/Al_2_O_3_, Mn/Al_2_O_3_) or lower temperature (350 °C) (Cu/Al_2_O_3_, Co/Al_2_O_3_). The highest hydrogen yield was found with the Fe/Al_2_O_3_ catalyst; also, increased catalyst Fe metal loading resulted in improved catalytic performance, with hydrogen yield increasing from 107 mmol g_plastic_^–1^ at 5 wt % Fe loading to 122 mmol g_plastic_^–1^ at 40 wt % Fe/Al_2_O_3_ Fe loading. Increased addition of steam to the (iii) water gas shift reactor in the presence of the Fe/Al_2_O_3_ catalyst resulted in higher hydrogen yield; however, as further steam was added, the hydrogen yield decreased due to catalyst saturation. The Fe-based catalyst support materials investigated alumina (Al_2_O_3_), dolomite, MCM-41, silica (SiO_2_), and Y-zeolite; all showed similar hydrogen yields of ∼118 mmol g_plastic_^–1^, except for the Fe/MCM-41 catalyst, which produced only 88 mmol g_plastic_^–1^ of hydrogen yield.

## 1 Introduction

Approximately 370 million tonnes of plastics are manufactured worldwide each year, and as key materials, they have applications in a wide range of industrial, transport, commercial, and domestic sectors.^1^ The use phase of plastic products ranges from <1 year, for example, for drinks bottles, to >50 years, for example, for home and building insulation.^2^ The end-of-life use of plastic products results in inevitable waste plastic production, and the United States and Europe represent major generators of waste plastics with annual tonnages of >40 and 29 Mt/y, respectively.^2,3^ There is widespread concern about the impact of waste plastics on the environment and the need for innovative solutions for improved waste plastic management.^4,5^ However, waste plastic recycling remains much lower than the amount of waste plastics going to landfill or waste incineration, both options representing a waste of resource. In addition, the vast majority of waste plastic recycling is through mechanical recycling to produce a recyclate material used to produce mainly low-grade products such as garden furniture, industry plastic pallets, fencing materials, traffic cones, waste bins, automotive parts, etc. Using more advanced recycling processes for waste plastics such as thermochemical pyrolysis or gasification can produce higher-value products such as liquid fuels, gasoline, chemicals, syngas, etc.^6−8^ Hydrogen is another high-value product that can be produced from waste plastics that has recently been under research investigation.^9−12^

The interest in hydrogen is that hydrogen is a major commodity chemical used extensively in petroleum refining, production of ammonia for fertilizer, and production of cyclohexane and methanol as feedstock for the plastics and pharmaceutical industries.^13^ In addition, hydrogen has been proposed as a major contributor to the decarbonization of the energy sector since it is regarded as a clean nonpolluting fuel and can be used in transport engines and fuel cells. Hydrogen is currently commercially produced almost exclusively from fossil fuels, natural gas methane (76%), and coal (23%), and the most widely used technology is through the catalytic steam reforming process.^13^ The steam reforming process using natural gas methane as a feedstock involves the reaction of high-temperature steam with the methane at high temperature (700–1000 °C) and pressure (0.3–2.5 MPa) in the presence of a nickel-based catalyst.^14^ The main reaction products are hydrogen and carbon monoxide
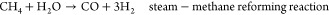
1To further improve the yield of hydrogen, in the commercial process, there is a downstream water gas shift reaction system where the carbon monoxide is catalytically converted to hydrogen and carbon dioxide
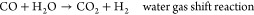
2In practice, the water gas shift reaction stage involves two separate and successive reactor stages with different operating temperatures and different metal catalysts to maximize the conversion to hydrogen. The first high-temperature water gas shift reactor operates in the temperature range of 310–450 °C with an iron-based catalyst followed by a low-temperature shift stage at between 200 and 250 °C with a copper-based catalyst. The final process step involves the removal of the carbon dioxide and other impurities by, for example, pressure swing adsorption to produce an essentially pure hydrogen end product.

Producing hydrogen from waste plastics would offer an alternative sustainable feedstock compared to that of fossil fuel natural gas or coal. There have been many studies in relation to the production of hydrogen from waste plastics based on the commercial catalytic steam reforming process but replacing natural gas with plastic pyrolysis gases. The process involves two-stage (i) plastic pyrolysis to produce volatile hydrocarbons followed by direct (ii) in-line catalytic steam reforming of the evolved plastic pyrolysis hydrocarbons to produce hydrogen and byproduct carbon monoxide.^15−19^ The process mimics the commercial natural gas catalytic steam reforming process, but reforming involves plastic pyrolysis gases consisting of hydrogen and a wide range of hydrocarbon gases, which can range from C_1_ to C_60_ for linear and branched hydrocarbon plastics such as polypropylene and polyethylene rather than methane.^20^ Inevitably, because plastic pyrolysis produces a wide range of hydrocarbon species, the reaction environment is complicated compared to the catalytic steam reforming of methane. Santamaria et al.^21^ have reviewed the process of pyrolysis-steam reforming of waste plastics (and biomass) with particular emphasis on the different catalysts used in the process. Nickel–alumina is highlighted as a catalyst used extensively in the studies of pyrolysis-catalytic steam reforming of waste plastics due to its relatively low cost, high activity of nickel metal, and the properties of the support material alumina of high surface area, strength, and stability.

Of further interest, and the focus of this report, is to add a third stage to the two-stage (i) pyrolysis and (ii) catalytic steam reforming process, consisting of a catalytic water gas shift reactor, to convert the carbon monoxide produced from the hydrocarbon steam reforming process to increase hydrogen production. The three-stage process further mimics the commercial process of natural gas to hydrogen process. Indeed, in an early pioneering study of pyrolysis-catalytic steam reforming of waste plastics reported by Czernik and French,^22^ the potential of an additional third-stage water gas shift reactor was proposed and was estimated that hydrogen yield from polypropylene could be increased by 12%.

The water gas shift reaction (eq 2) is exothermic (reaction enthalpy of −41 kJ mol^–1^) and reversible, indicating that the forward reaction to produce hydrogen is thermodynamically promoted at lower temperature and kinetically promoted at higher temperature. For example, Mendes et al.^23^ reported on the influence of temperature on CO conversion to produce hydrogen in a steam reforming water gas shift reactor and showed that increasing reaction temperature reduces the equilibrium conversion of CO. Hence, two water gas shift reactors, high temperature (310–450 °C) and low temperature (200–250 °C), exist for the commercial production of hydrogen from natural gas. However, in this work, we have used a single, third-stage water gas shift reactor operated at a controlled single temperature, where the temperature of the third-stage catalytic water gas shift reactor was an investigated process variable. The commercial two-step high- and low-temperature water gas shift reactors also use two different types of catalysts, iron-based and copper-based catalysts, respectively. Pal et al.^24^ have reviewed the different types of catalysts used in the water gas shift reaction. The iron-based catalysts used for high-temperature shift reactions are based on Fe with the presence of (Cr_2_O_3_ oxide) to stabilize the catalyst and prevent sintering, typically in the form of Fe_2_O_3_, which is reduced to Fe_3_O_4_ during the reaction as the active phase. Metal promoters have been added to the Fe/Cr catalyst to improve catalyst effectiveness, for example, the addition of Cu to prevent methanation of the CO and improve selectivity for H_2_ or replacement of toxic Cr with Ce.^24^ Low-temperature copper (CuO)-based catalysts typically also contain Al_2_O_3_/Cr_2_O_3_ and ZnO, which provide structural support and minimize sulfur poisoning. Metal promoters added to the copper-based catalysts have included Mn and Ni. It has also been shown that the steam input to the water gas shift reactor influences the CO equilibrium conversions.^23^

In this work, we report on the three-stage (i) pyrolysis, (ii) catalytic steam reforming, and (iii) water gas shift processing of waste plastics for the production of hydrogen. The (i) pyrolysis and (ii) catalytic steam reforming process conditions are maintained throughout the experimental program, and we investigate the process conditions of the (iii) water gas shift reactor. The influence of different metal–alumina catalysts (Co, Cu, Zn, Mn, and Fe) in relation to catalyst temperature is investigated with the aim of identifying an effective catalyst for the waster gas shift reaction operating at a single-stage temperature. In addition, different process conditions in the third-stage (iii) water gas shift reactor of the amount of catalyst metal loading (Fe), steam input flow rate, and type of catalyst support material are also investigated.

## 2 Materials and Methods

### 2.1 Materials

The polypropylene was supplied by Beijing Ou Yuan Sheng Plastic Production Co., Ltd., and was in the form of 2 mm pellets. A Thermos EA-2000 elemental analyzer was used to complete an elemental analysis of the polypropylene plastic feedstock and showed a carbon content of 84.45 wt %, hydrogen of 13.81 wt %, nitrogen of 0.03 wt %, oxygen of 0.91, and sulfur 0.07. A Shimadzu TGA-50 instrument was used to complete a proximate analysis of the plastic sample and showed a volatile content of 98.70 wt % and an ash content of 0.79 wt %. The presence of ash, nitrogen, sulfur, and oxygen in the polypropylene sample is due to the sample being a postconsumer, “real-world” plastic waste sample, which may contain contaminants, other plastics, and plastic additives. For example, the presence of oxygen-containing plastic and poly(ethylene terephthalate) or the presence of additives introduced during the plastic manufacturing process to improve the properties of the plastic tailored to the end-use application such as antioxidants, UV absorbers, inorganic fillers, etc., may “contaminate” the polypropylene sample.

The influence of a catalyst support material was also investigated and involved alumina, silica (SiO_2_), and Y-zeolite obtained from Alfa Aesar Ltd., U.K., and dolomite (CaMg(CO_3_)_2_), MCM-41, and Y-zeolite obtained from Sigma-Aldrich, Ltd., U.K.

### 2.2 Catalyst Preparation

Catalysts were used in the second-stage (ii) reforming reactor and the third-stage (iii) water gas shift reactor. The catalyst employed in the second-stage (ii) steam reforming reactor was a 10 wt % Ni/Al_2_O_3_ catalyst, chosen due to its effective performance as both a reforming and cracking catalyst for the catalytic steam reforming of light and heavy hydrocarbons.^25,26^ As noted before, Ni/Al_2_O_3_ is commonly used in pyrolysis-catalytic steam reforming studies related to waste plastics.^21^ Although it has also been reported that there are disadvantages of using Ni/Al_2_O_3_ in that it is prone to coke formation due to the acidic nature of the material and there is much research into other support materials.^19^ Alternate transition metals, noble metals, and bimetallic catalysts in addition to metal promoters have also been investigated, as reviewed extensively by Santamaria et al.^21^ However, in this work, we have concentrated on maintaining constant conditions in the second-stage (ii) catalytic steam reforming stage and used a 10 wt % Ni/Al_2_O_3_ catalyst. In a later paper, we will report on the three-stage (i) pyrolysis, (ii) catalytic steam reforming, and (iii) water gas shift process while maintaining conditions and catalysts in the (iii) water gas shift stage and investigating a wide range of process parameters in the (ii) catalytic steam reforming stage. The 10 wt % Ni/Al_2_O_3_ catalyst was prepared using a wet impregnation technique by mixing the nickel nitrate hexahydrate precursor salt in 25 mL of distilled water for 30 min followed by the appropriate quantity of alumina and allowing a further 30 min of mixing. The slurry was mixed and heated at a heating rate of 1 °C min to 100 °C until a semisolid formed before being dried for 24 h at 105 °C in an oven. The catalyst was then calcined at a temperature of 750 °C in a furnace and then crushed and classified into 50–212 μm sized particles. The prepared catalysts were reduced at 800 °C for 2 h in the presence of hydrogen (5 wt % H_2_, 95 wt % N_2_).

The catalysts used in the third-stage (iii) water gas shift reactor consisted of 10 wt % of metal-loaded alumina catalysts, Fe/Al_2_O_3_, Zn/Al_2_O_3_, Cu/Al_2_O_3_, Co/Al_2_O_3_, and Mn/Al_2_O_3_, and were prepared also using a wet impregnation method. The metals chosen were based on catalysts known to be effective for hydrogen production at high temperature, i.e., Fe, and effective at low temperature (Cu),^23,24^ together with other transition metals for comparison to determine their effectiveness for processing waste plastics in the three-stage reactor system (Zn, Mn, Co). The metal salts used were Fe(NO_3_)_3_·9H_2_O (VWR Ltd.), Zn(NO_3_)_2_·6H_2_O (Sigma-Aldrich, U.K., Ltd.), Cu(NO_3_)_2_·2.5H_2_O (Alfa Aesar Ltd.), Co(NO_3_)_2_·6H_2_O (Acros Organics Ltd.), and Mn(NO_3_)_2_·4H_2_O (Sigma-Aldrich Ltd.). The wet impregnation process was exactly the same as described for the preparation of the 10 wt % Ni/Al_2_O_3_ catalyst used in the second-stage (ii) reforming reactor; however, the calcination temperature used for the third-stage (iii) water gas shift catalysts was 700 °C. The catalysts were reduced as before at 800 °C in the presence of hydrogen.

### 2.3 Three-Stage (i) Pyrolysis, (ii) Reformer, and (iii) Water Gas Shift Reactor System

**Figure 1:**
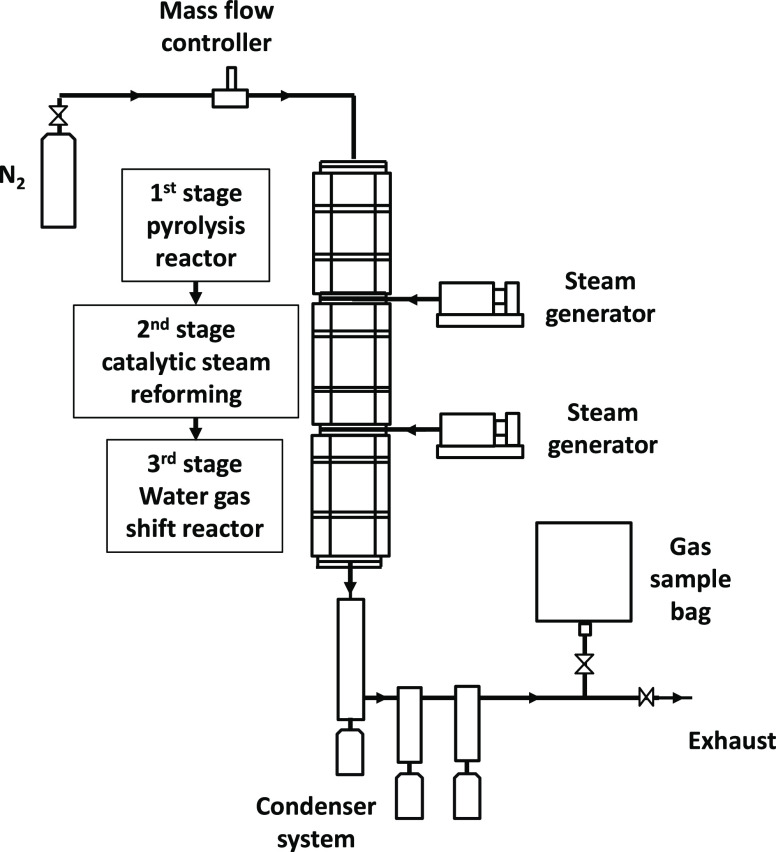
Schematic diagram of the three-stage pyrolysis-catalytic water gas shift fixed-bed reactor system.

Figure 1 shows a schematic diagram of the three-stage (i) pyrolysis, (ii) catalytic steam reformer, (iii) and water gas shift reactor system used to produce hydrogen from waste polypropylene. The first-stage (i) pyrolysis took place in a stainless steel reactor, 30 cm in length and 2.5 cm in diameter, and heated externally by a temperature-controlled electrically heated furnace. The polypropylene (1.00 g) was loaded into a stainless steel crucible, which was held in place in the center of the pyrolysis reactor. The pyrolysis heating regime consisted of heating the reactor from 20 to 500 °C at 20 °C min^–1^ and held at that temperature for 20 min. The evolved pyrolysis hydrocarbons derived from the thermal degradation of the polypropylene pyrolysis were passed directly to the reforming reactor where catalytic steam reforming took place in the presence of the 10 wt % Ni/Al_2_O_3_ catalyst (1.00 g). The reforming reactor was also constructed of stainless steel (length 30 cm, 2.5 cm diameter) heated with a temperature-controlled electrical furnace. The steam required for reforming was supplied to the second-stage reactor via a water syringe pump (WPI SPLG100 syringe pump) to give a controlled input of steam. The temperature of the second-stage (ii) reforming catalytic reactor was maintained at 850 °C throughout the experiments.

The product gases from the second-stage (ii) reforming reactor were passed directly to the third-stage stainless steel (iii) water gas shift reactor, of length 14.5 cm and 2 cm diameter, heated by a temperature-controlled furnace. The product gases from the reforming reactor consist of mainly hydrogen and carbon monoxide undergoing catalytic water gas shift reaction in the presence of the metal–alumina catalysts (0.50 g). Steam was generated from water added via a second WPI SPLG100 syringe pump. The temperature of the water gas shift catalytic reactor was an investigated process parameter, and temperatures between 250 and 650 °C were examined using each of the different metal–alumina (Fe, Zn, Mn, Cu, Co) catalysts. Thermocouples monitored the temperatures of the pyrolysis, catalytic steam reforming, and catalytic water gas shift processes throughout the experiments. The three-stage reactor system was continually purged with nitrogen at 100 mL min^–1^, producing a nominal gas residence time of 88 s in the second-stage (ii) reforming reactor and 26 s in the third-stage (iii) water gas shift reactor. The gases leaving the reactor system were passed through a series of water and dry-ice-cooled glass condensers to remove condensable products, which consisted of mostly unreacted water (condensed steam). The final product gases were collected in a 25 L Tedlar gas sample bag.

The experimental procedure for the operation of the three-stage reactor system involved initially heating the (ii) second- and (iii) third-stage catalytic reactors to the desired temperature, 850 °C for the (ii) second stage and investigated temperatures between 250 and 650 °C for the (iii) third stage. Once the catalyst reactor temperature had been stabilized, the pyrolysis reactor was heated to 500 °C at 20 °C min^–1^ and held at that temperature for 20 min. The heating of the first-stage (i) pyrolysis coincided with the injection of steam into the (ii) reformer reactor and the third-stage (iii) water gas shift reactor. The reactor system was tested via many baseline experiments to determine repeatability and reproducibility, and only negligible differences occurred between experiments. Data reported here were the average of at least two repeat experiments.

### 2.4 Gas Analysis

Gas analysis of the product gases in the gas sample bag was carried out immediately after each experiment using packed column gas chromatography (GC). A Varian CP-3330 gas chromatograph (Varian U.K., Ltd.) equipped with a HayeSep 60–80 mesh column, 2 m in length and 2 mm diameter with an Ar carrier gas and a thermal conductivity detector (TCD) was used to detect the relative quantities of the permanent gases: H_^2^_, N_2_, O_2_, and CO. Due to the similar retention times of CO and CO_2_, the quantity of CO_2_ was determined on a separate Varian CP-3330 GC operating with the same conditions but a finer mesh column (80–100 mm). A third Varian CP-3380 GC was used to analyze hydrocarbons gases (C_1_–C_4_) with 2 m in length and 2 mm in diameter HayeSep packed column (80–100 mm mesh), N_2_ carrier gas, and flame ionization detector (FID). Calibration of the gas chromatographs used standard mixtures of 1% permanent and hydrocarbon gases. The mass of each gas was calculated from the volumetric gas chromatographic data, known N_2_ gas flow rate, properties of each gas, and the ideal gas law. The total mass of gas and hydrogen yield produced from polypropylene was then determined from eq 3 to 4
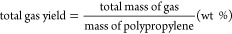
3
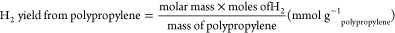
4

### 2.5 Catalyst Analysis

The freshly prepared metal–alumina catalysts used in the third-stage (iii) water gas shift reactor were analyzed for their surface area (Brunauer–Emmett–Teller (BET)) and pore volume determined using a Nova-2200e instrument. The catalyst was held under vacuum at 77 K before being exposed to a flow of N_2_ at various pressures; the degree to which N_2_ is adsorbed can be used to determine the surface area and porosity of the sample. In addition, the crystallinity and metal particle size of the freshly prepared metal–alumina catalysts were determined with a Bruker D-8 diffractometer using a Cu Kα X-ray source operating at 40 kV and 40 mA with a Vantec position-sensitive detector. The spectra were analyzed with a database of known spectra to identify the peaks. The crystallite particle size was also calculated using High Score Plus software with a built-in function for the Scherrer equation (crystal particle size) calculation.

Characterization of both the fresh and spent catalyst samples was completed to understand the morphology of the catalysts and to determine the dispersion of the metals on the catalyst surface. A Hitachi SU8230 scanning electron microscope operated at 2.0 kV with an Oxford Instruments Aztec Energy EDXS (energy-dispersive X-ray spectrometry) system was used to produce high-resolution images and to identify the elements present in the sample. The catalyst samples were placed on an aluminum stub and coated with carbon and then 10 nm iridium/platinum coating (to counteract negative charges under the microscope).

The catalysts after use in the pyrolysis-reforming water gas shift reactor system were analyzed by temperature-programmed oxidation (TPO) to identify the amount of carbon deposition (coking) present on the catalyst using a Shimadzu TGA-50 thermogravimetric analyzer. The catalyst sample was heated at 20 °C min^–1^ from ambient conditions to 800 °C in an atmosphere of air with a flow rate of 50 mL min^–1^. The weight loss of the catalyst due to the oxidation of the carbon deposits was determined in relation to the increase in temperature.

## 3 Results and Discussion

### 3.1 Characteristics of the Freshly Prepared Catalysts

Table 1 shows the BET surface area and pore volume of the different 10 wt % metal–alumina catalysts used in the third-stage (iii) water gas shift reactor. The surface area ranged from 89 m^2^ g^–1^ for the Cu/Al_2_O_3_ and Co/Al_2_O_3_ catalysts to 156 m^2^ g^–1^ for the Mn/Al_2_O_3_ catalyst. The surface area of the fresh catalyst was in the order Mn/Al_2_O_3_ > Zn/Al_2_O_3_ > Fe/Al_2_O_3_ > Cu/Al_2_O_3_ ≥ Co/Al_2_O_3_. The Cu/Al_2_O_3_ and Co/Al_2_O_3_ catalysts also showed the lowest pore volume compared to the Mn/Al_2_O_3_, Zn/Al_2_O_3_, and Fe/Al_2_O_3_ catalysts, which were significantly higher.

**Table 1:** BET Surface Area and Pore Volume of 10 wt % of Different Metal–Alumina Catalysts

catalyst	BET surface area (m^2^ g^–1^)	pore volume (cm^3^ g^–1^)
10% Fe/Al_2_O_3_	134	0.3602
10% Zn/Al_2_O_3_	151	0.3757
10% Mn/Al_2_O_3_	156	0.3732
10% Cu/Al_2_O_3_	89	0.2137
10% Co/Al_2_O_3_	89	0.1943

**Figure 2:**
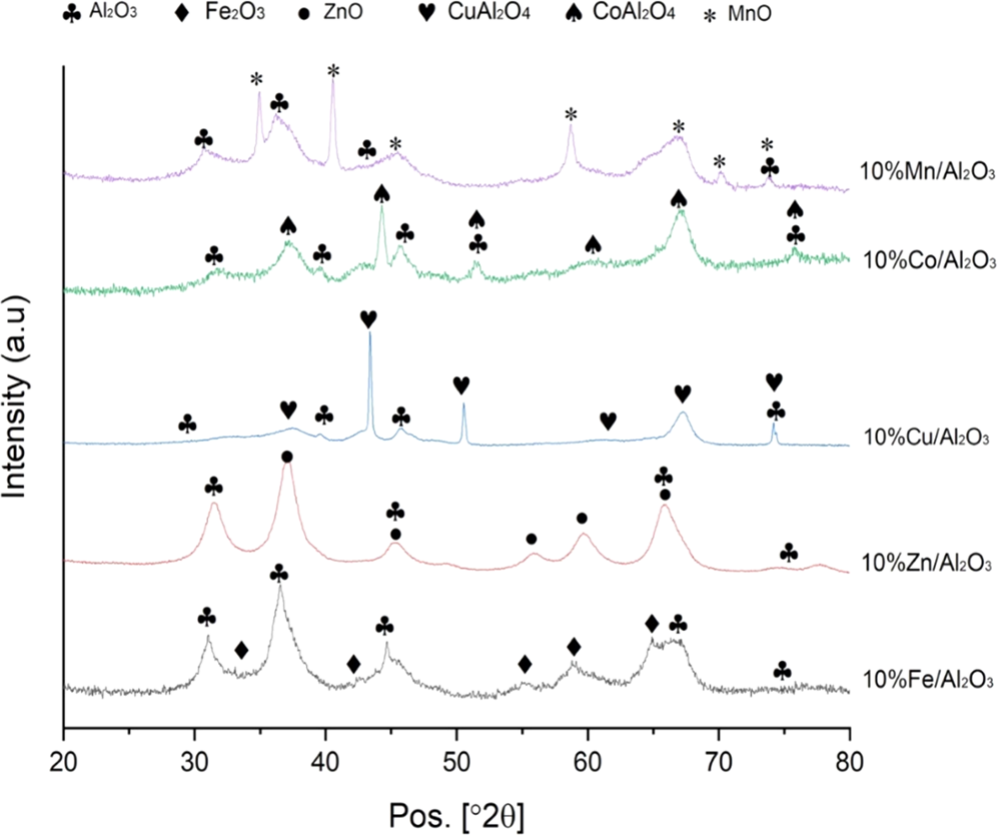
XRD spectra of the different metal–alumina catalysts used in the third-stage (iii) water gas shift reactor.

The freshly prepared catalysts analyzed by X-ray diffraction (XRD) enabled the determination of the crystalline phases of the catalysts. The results showed that the Fe_2_O_3_ phase was identified in the Fe/Al_2_O_3_ catalyst, a ZnO phase was present in the Zn/Al_2_O_3_ catalyst, CuAl_2_O_4_ was present in the Cu/Al_2_O_3_ catalyst, CoAl_2_O_4_ spinel was identified in the Co/Al_2_O_3_ catalyst, and MnO was present in the Mn/Al_2_O_3_ catalyst (Figure 2). The XRD data were used to calculate the average crystal size of the catalysts using the Scherrer equation (eq 5)
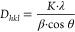
5where *K* is a dimensionless shape factor (where *K* = 0.89 where β is line broadening at half the maximum intensity (FWHM)) and λ is the X-ray wavelength (0.154056 nm) with a scanning step of 0.033° obtained using Cu Ka radiation. The diffraction angles used were 58.6, 59, 44.5, 45, and 43 for the Fe/Al_2_O_3_, Zn/Al_2_O_3_, Cu/Al_2_O_3_, Co/Al_2_O_3_, and Mn/Al_2_O_3_ catalysts, respectively. The determined average crystal sizes were 21.7, 16.8, 43.1, 21.2, and 29.4 nm for the Fe/Al_2_O_3_, Zn/Al_2_O_3_, Cu/Al_2_O_3_, Co/Al_2_O_3_, and Mn/Al_2_O_3_ catalysts, respectively.

**Figure 3:**
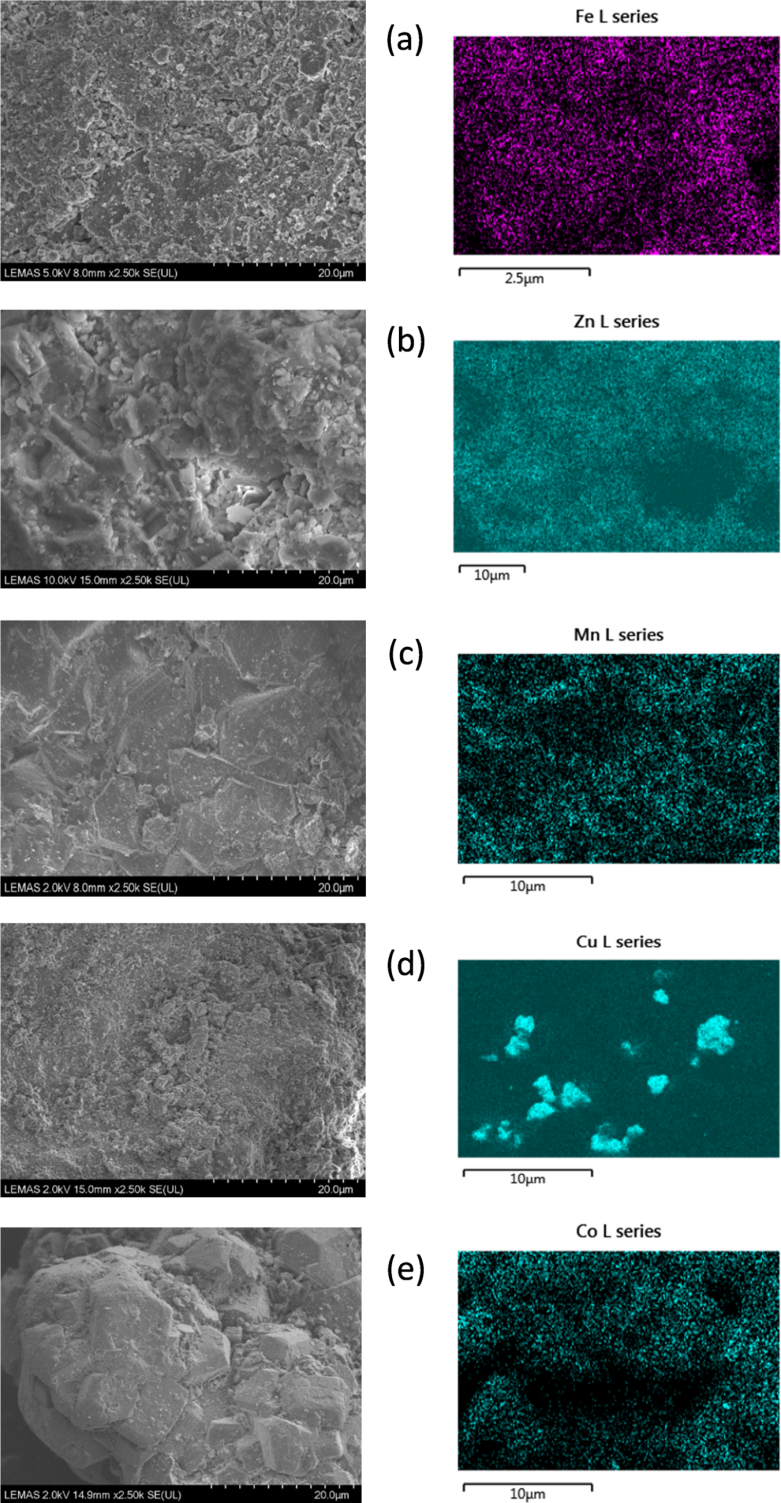
SEM–EDXS analysis of different metals on alumina support catalysts: (a) 10 wt % Fe/Al_2_O_3_, (b) 10 wt % Zn/Al_2_O_3_, (c) 10 wt % Mn/Al_2_O_3_, (d) 10 wt % Cu/Al_2_O_3_, and (e) 10 wt % Co/Al_2_O_3_.

Scanning electron microscopy coupled with the catalyst surface mapping of the active metal species (SEM–EDXS) was used to explore the morphology of the catalysts and distribution of metal on the alumina support. The results are shown in Figure 3 and show that the metal particles were more uniformly dispersed in the Fe/Al_2_O_3_, Zn/Al_2_O_3_, Mn/Al_2_O_3_, and Co/Al_2_O_3_ catalysts; however, sintering and nonuniform metal particle distribution were observed with the Cu/Al_2_O_3_ catalyst.

### 3.2 Hydrogen Production from the Three-Stage Processing of Polypropylene

Initial work investigated the hydrogen production from the processing of polypropylene using a single (i) pyrolysis stage, a two-stage (i) pyrolysis and (ii) catalytic reforming reactor system, and a three-stage (i) pyrolysis, (ii) catalytic reforming, and (iii) water gas shift reactor system. The process conditions for (i) pyrolysis were heated from 20 to 500 °C at 20 °C min^–1^, and then the reactor was held at 500 °C for 20 min. The hydrogen yield from the pyrolysis of polypropylene was 72.7 mmol g_plastic_^–1^. However, when the second-stage (ii) catalytic reforming reactor was added to the (i) pyrolysis stage to produce a two-stage (i) pyrolysis and (ii) catalytic reforming stage, the hydrogen yield was significantly increased to 106.7 mmol g_plastic_^–1^. For the two-stage reactor system, the catalyst was a 10 wt % Ni/Al_2_O_3_ catalyst held at 850 °C and the steam input was 4 mL h^–1^. The markedly increased hydrogen yield showed the effectiveness of the catalytic steam reforming of the volatiles produced from the pyrolysis of polypropylene. When the third-stage shift reactor was added to produce a three-stage pyrolysis, (ii) catalytic steam reforming, and (iii) water gas shift reactor system, the hydrogen yield was further increased to 115.8 mmol g_plastic_^–1^. The process conditions for the three stages were pyrolysis at 500 °C, catalytic steam reforming at 850 °C with the 10 wt % Ni/Al_2_O_3_ catalyst and steam input 4 mL h^–1^ and for the water gas shift reactor, 550 °C catalyst temperature with a 10 wt % Fe/Al_2_O_3_ catalyst and 4 mL h^–1^ steam input. This preliminary work suggests that the three-stage reactor system incorporating a third-stage water gas shift reactor can aid the improvement of hydrogen yield by the reaction of steam and carbon monoxide via the water gas shift reaction. A further investigation of the process conditions and catalysts used in the third-stage water gas shift reactor was therefore undertaken with the aim of improving the hydrogen yield from the processing of polypropylene.

**Figure 4:**
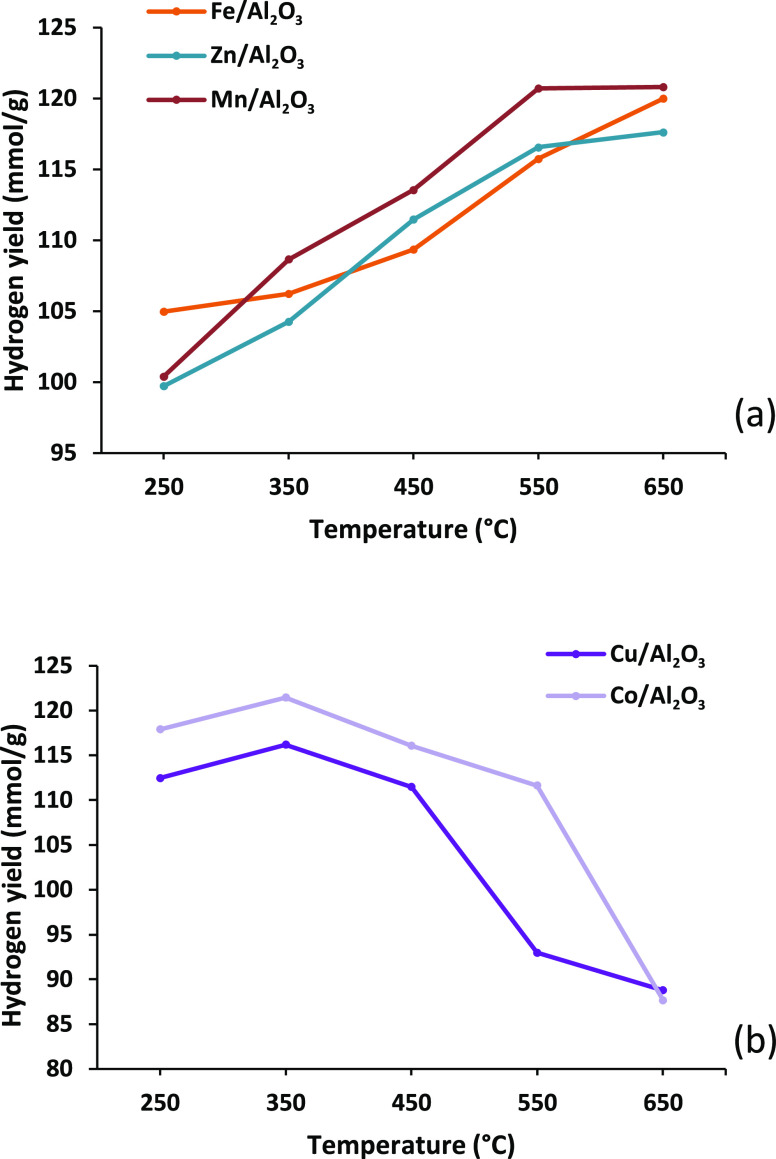
Hydrogen yield (mmol g_plastic_^–1^) for the three-stage (i) pyrolysis, (ii) catalytic steam reforming (10 wt % Ni/Al_2_O_3_), and (iii) water gas shift reaction process using different monometallic alumina catalysts in relation to catalyst temperature. Catalysts presented in terms of (a) catalysts effective at high temperature and (b) catalysts effective at low temperature.

The influence of catalyst temperature in the third-stage (iii) water gas shift reactor in relation to different metal-based catalysts at temperatures of 250, 350, 450, 550, and 650 °C was investigated. The other experimental conditions were maintained at a pyrolysis final temperature of 500 °C, a catalytic steam reforming temperature of 850 °C with a 10 wt % Ni/Al_2_O_3_ catalyst, with steam (water) introduced to each of the second and the third stages with an input flow rate of 4 mL h^–1^. Figure 4 shows the influence of temperature on the yield of hydrogen from the pyrolysis-catalytic steam reforming water gas shift processing of the polypropylene. For clarity, the results have been separated into those that have higher hydrogen yield at high temperature (Fe/Al_2_O_3_, Zn/Al_2_O_3_, Mn/Al_2_O_3_) and those giving higher hydrogen yield at lower temperature (Cu/Al_2_O_3_, Co/Al_2_O_3_). Figure 4 shows that the catalysts investigated show distinct differences with clear higher yields at either high temperature or low temperature. Chen et al.^27^ reported that water gas shift catalysts could be categorized into either high or low temperature depending on how they interact with the carbon monoxide and steam to produce hydrogen via the water gas shift reaction.

The exothermic nature of the water gas shift reaction would suggest that a decrease in CO conversion would occur as the temperature was increased. However, since the reaction rate is temperature-dependent, a competing kinetic reaction can result in an increase in CO conversion as the temperature is increased. The use of distinct low-temperature and high-temperature water gas shift catalysts commercially is due to the different extents of reduction in activation energy. Chen et al.^27^ conducted experiments into the water gas shift reaction using low- and high-temperature reactors with low- and high-temperature catalysts Cu–Zn and Fe–Cr catalysts, respectively. They reported that an increase in temperature from 200 to 400 °C with the Cu–Zn-catalyzed low-temperature reactor resulted in a decrease in CO conversion, which was attributed to the exothermic nature of the water gas shift reaction and Le Chatelier’s principle dominating. However, increasing the temperature from 300 to 500 °C with the Fe–Cr catalyst in the high-temperature reactor showed a distinct increase in CO conversion, in this case attributed to the reaction kinetics dominating.^27^ The results here also show that the high-temperature catalysts, Fe, Zn, and Mn, have positive correlations between hydrogen production and temperature, but the low-temperature catalysts, Cu and Co, have negative correlations between hydrogen production and increased temperature. However, all of the metal–alumina catalysts used in this investigation have catalyzed the water gas shift reaction and increased the hydrogen production but to different extents. Iron-based catalysts are the most common high-temperature catalysts used commercially.^24^ However, the Zn and Mn catalysts along with the Fe catalyst all produced about 120 mmol g_plastic_^–1^ of hydrogen, indicating that both Zn and Mn are potentially equally as active as the commonly used Fe catalyst for the water gas shift process. Figure 4b shows the influence of increasing catalyst temperature for the low-temperature Cu and Co catalysts in the third-stage (iii) water gas shift reactor on the yield of hydrogen from the pyrolysis-catalytic steam reforming water gas shift processing of the polypropylene. Increasing the catalyst temperature from 250 to 650 °C results in an initial increase in hydrogen yield to 115–120 mmol g_plastic_^–1^ at a 350 °C catalyst temperature but was followed by a marked decrease in hydrogen yield to ∼90 mmol g_plastic_^–1^ as the temperature was further increased to 650 °C. Cu-based catalysts are the most commonly used and effective catalysts used commercially for the low-temperature water gas shift reaction.^28^ In this work, the Co/Al_2_O_3_ catalyst produced a higher yield of hydrogen compared to the Cu/Al_2_O_3_ catalyst. However, the Cu-based catalysts used commercially generally also include ZnO as a structural support and minimize sintering.^28^

**Figure 5:**
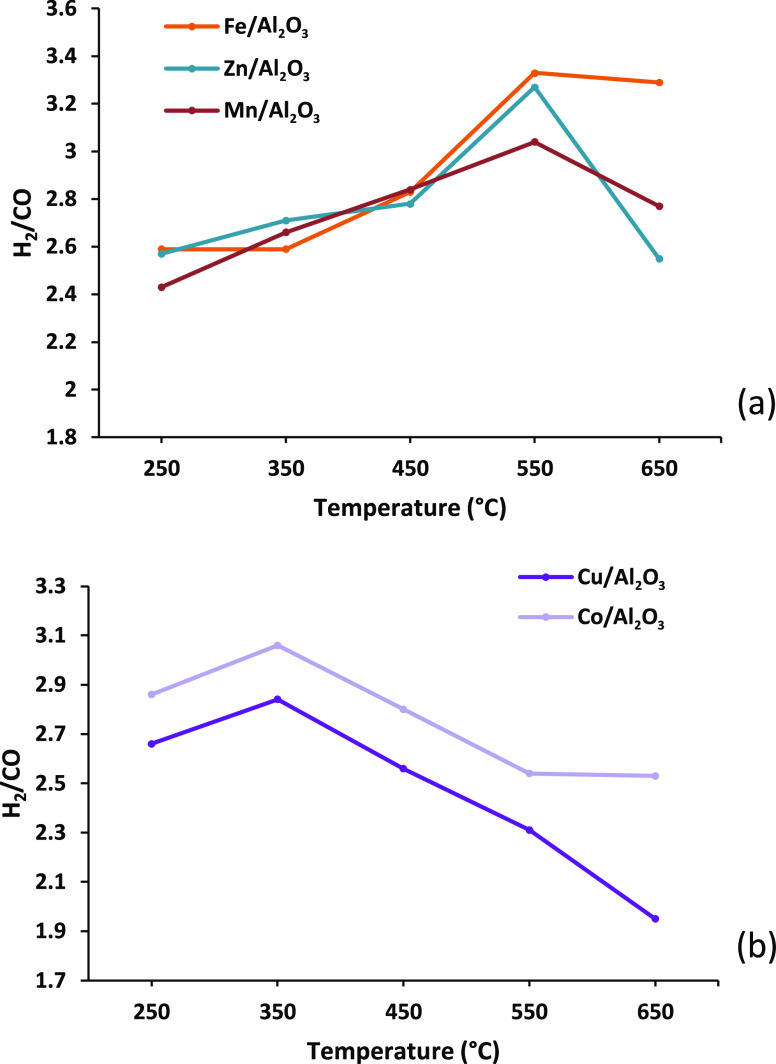
Hydrogen/carbon monoxide molar ratio for the three-stage (i) pyrolysis, (ii) catalytic steam reforming (10 wt % Ni/Al_2_O_3_), and (iii) water gas shift reaction process using different monometallic alumina catalysts in relation to catalyst temperature. Catalysts presented in terms of (a) catalysts effective at high temperature and (b) catalysts effective at low temperature.

Figure 5 shows the hydrogen/carbon monoxide molar ratio for the three-stage (i) pyrolysis, (ii) catalytic steam reforming, and (iii) water gas shift reaction process using the different monometallic catalysts in relation to catalyst temperatures. The data are presented in terms of the catalysts effective at high temperature (Fe, Zn, Mn) and the catalysts effective at low temperature (Cu, Co). Figure 5a shows the H_2_/CO ratios for the Fe, Zn, and Mn, high-temperature catalysts and shows that the highest ratios, and therefore the most effective temperature, were reached at a catalyst temperature of 550 °C, coinciding with the catalyst temperature for the highest hydrogen yield. The Fe/Al_2_O_3_ catalyst achieved a H_2_/CO ratio of 3.33, while Zn and Mn achieved H_2_/CO ratios of 3.27 and 3.04, respectively. As the catalyst temperature was increased to 650 °C, the Fe/Al_2_O_3_ catalyst maintained this high H_2_/CO ratio, whereas the Zn/Al_2_O_3_ and Mn/Al_2_O_3_ catalysts showed a marked decrease. Figure 5b shows the results of the product H_2_/CO ratio for the catalysts shown to be effective at low temperature in this work (Cu, Co). The results for Co and Cu show the highest H_2_/CO ratio at a catalyst temperature of 350 °C, which coincides with the catalyst temperature for peak hydrogen production.

**Figure 6:**
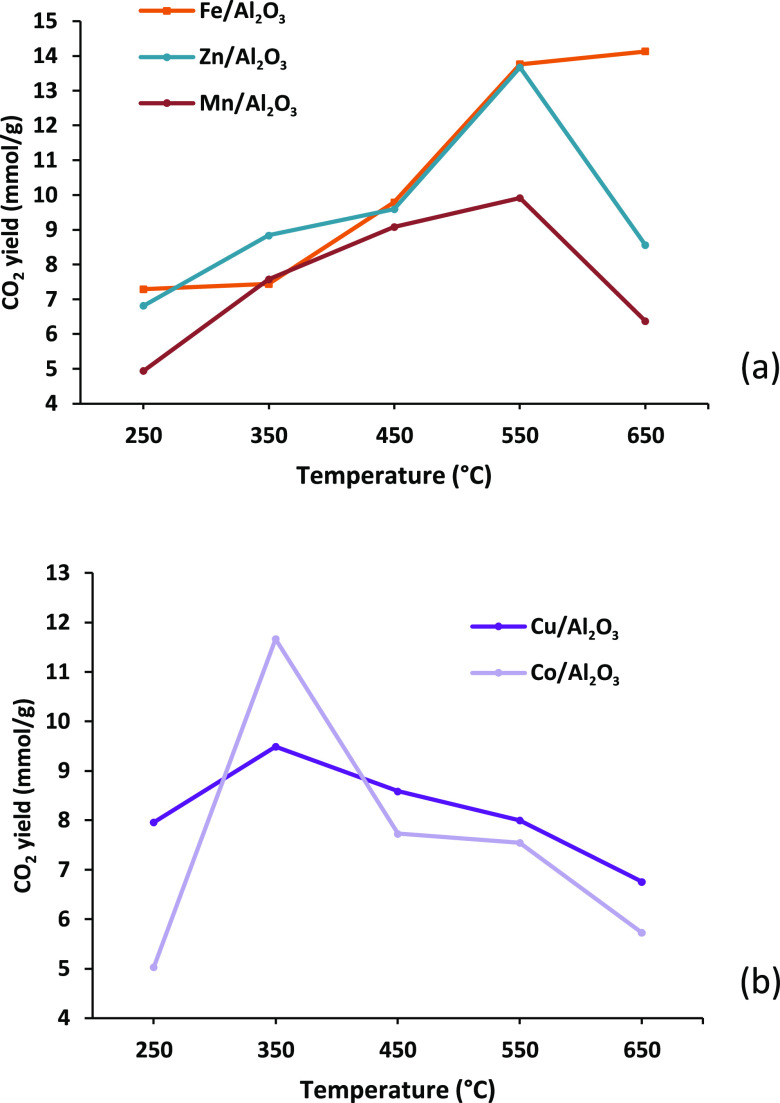
Carbon dioxide yield (mmol g_plastic_^–1^) for the three-stage (i) pyrolysis, (ii) catalytic steam reforming (10 wt % Ni/Al_2_O_3_), and (iii) water gas shift reaction process using different monometallic alumina catalysts in relation to catalyst temperature. Catalysts presented in terms of (a) catalysts effective at high temperature and (b) catalysts effective at low temperature.

A byproduct of the production of hydrogen from the water gas shift reaction of steam with carbon monoxide is carbon dioxide. Figure 6 shows the yield of carbon dioxide for the three-stage processing of polypropylene in relation to the different metal–alumina catalysts in the (iii) third stage in relation to catalyst temperature. Figure 6a shows the catalysts effective at high temperature, and Figure 6b shows the catalysts effective at low temperature. The yield of CO reflects the yield of hydrogen shown in Figure 4, with the high-temperature catalysts (Fe, Zn, Mn) showing a peak of CO_2_ yield at a catalyst temperature of 550 °C, which is the same as the peak of hydrogen production, reflecting the effectiveness of the water gas shift reaction in the third-stage reactor. Similarly, the yield of CO_2_ for the low-temperature catalysts (Cu, Co) peaked at a catalyst temperature of 350 °C, which reflected the peak yield of hydrogen.

**Figure 7:**
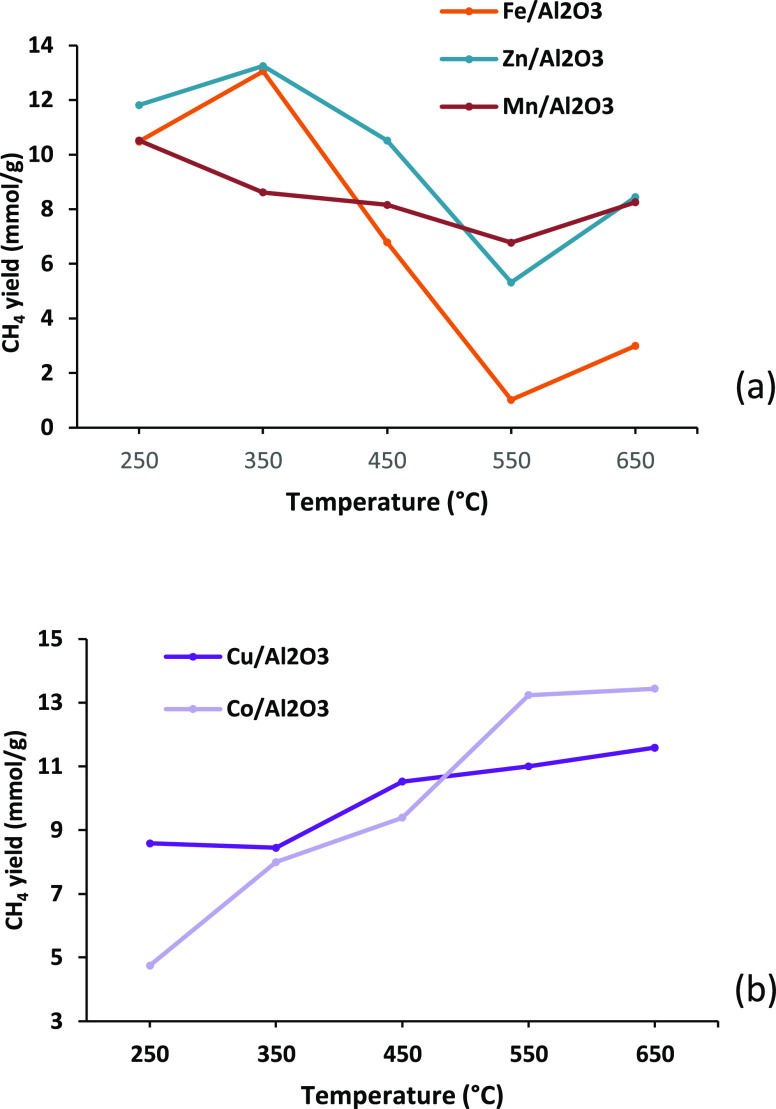
Methane yield (mmol g_plastic_^–1^) for the three-stage (i) pyrolysis, (ii) catalytic steam reforming (10 wt % Ni/Al_2_O_3_), and (iii) water gas shift reaction process using different monometallic alumina catalysts in relation to catalyst temperature. Catalysts presented in terms of (a) catalysts effective at high temperature and (b) catalysts effective at low temperature.

A key component in evaluating the overall efficiency of the water gas shift catalyst is the selectivity in relation to the reaction since the generation of methane from carbon monoxide and hydrogen (methanation) is an undesirable side reaction that may occur in the process. Therefore, it is important to compare the production of CH_4_ to the H_2_/CO ratios since the ratio might change due to the methanation reaction, which decreases the CO content and so increases the value of H_2_/CO. Figure 7 shows the methane yield for the three-stage processing of polypropylene using the different (iii) water gas shift stage monometallic alumina catalysts in relation to catalyst temperature. The high-temperature catalysts operated at a temperature of 550 °C with the Mn and Zn catalysts produced a CH_4_ yield of 6.78 and 5.32 mmol g_plastic_^–1^, respectively, which is higher than the 1.02 mmol g_plastic_^–1^ produced by the Fe catalyst. This demonstrates that Fe is highly selective toward the water gas shift reaction, while both Zn and Mn appear to favor methanation and exhibit no inhibition of activity at temperatures higher than 550 °C.

Temperature-programmed oxidation (TPO) of the spent metal–alumina catalysts used for the production of hydrogen from the three-stage processing of polypropylene was undertaken to determine the extent of coke deposition on the catalyst. The issue of the formation of coke on the catalysts is a major disadvantage of the catalyst activity, which causes deactivation.^29^ The TPO was determined using a thermogravimetric analyzer, which combusted the carbon in an air atmosphere and the weight loss corresponding to the mass of carbonaceous coke on the catalyst. The results showed that the mass of coke deposited was 3.7 wt % for the Fe/Al_2_O_3_ catalyst, 3.9 wt % for the Zn/ Al_2_O_3_, 3.7 wt % for Cu/ Al_2_O_3_>, 4.1 wt % for Co/ Al_2_O_3_>, and 6.1 wt % for the Mn/Al_2_O_3_ catalyst. Therefore, the amount of coke deposited on the catalysts showed no correlation with the high- or low-temperature nature of the water gas shift catalyst used.

### 3.3 Bimetallic Catalysts for Hydrogen Production from the Three-Stage Processing of Polypropylene

As previously stated, the commercial production of hydrogen from natural gas catalytic steam reforming involves downstream separate high-temperature and low-temperature water gas shift catalytic reactors to enhance hydrogen production. However, in this work, only a single-stage reactor was used to investigate the production of hydrogen from the three-stage processing of polypropylene. The previous results have shown clearly the benefits of having two water gas shift reactors, operating at high temperature and low temperature with specific catalysts effective for enhancing the water gas shift reaction to maximize hydrogen production. The aim of the research presented in this section was to produce a bimetallic catalyst using a combination of the metals shown to be effective at high temperature with metals shown to be effective at low temperature and operating the reactor at a single temperature. The maximum hydrogen yield using the high-temperature Fe, Zn, and Mn catalysts was obtained at a catalyst temperature of 550 °C, whereas for the low-temperature catalysts, Cu and Co, the maximum hydrogen yield was obtained at a catalyst temperature of 350 °C. In addition, the highest overall hydrogen yield was obtained with the Fe/Al_2_O_3_ catalyst. Therefore, Fe was used as the main catalyst and Zn, Co, and Cu were added to the Fe to produce bimetallic catalysts composed of Fe–Zn, Fe–Cu, Fe–Co, and Fe–Mn. The wt % of metal was 5 wt %, which produced overall 10 wt %, for example, 5 wt % of Fe with 5 wt % of Zn/Al_2_O_3_. The single operating catalyst temperature used was 350 °C. The experimental conditions were therefore maintained at a pyrolysis final temperature of 500 °C, a catalytic steam reforming temperature of 850 °C, and a water gas shift catalyst temperature of 350 °C with steam (water) introduced to each of the second and the third stages with an input flow rate of 4 mL h^–1^.

**Figure 8:**
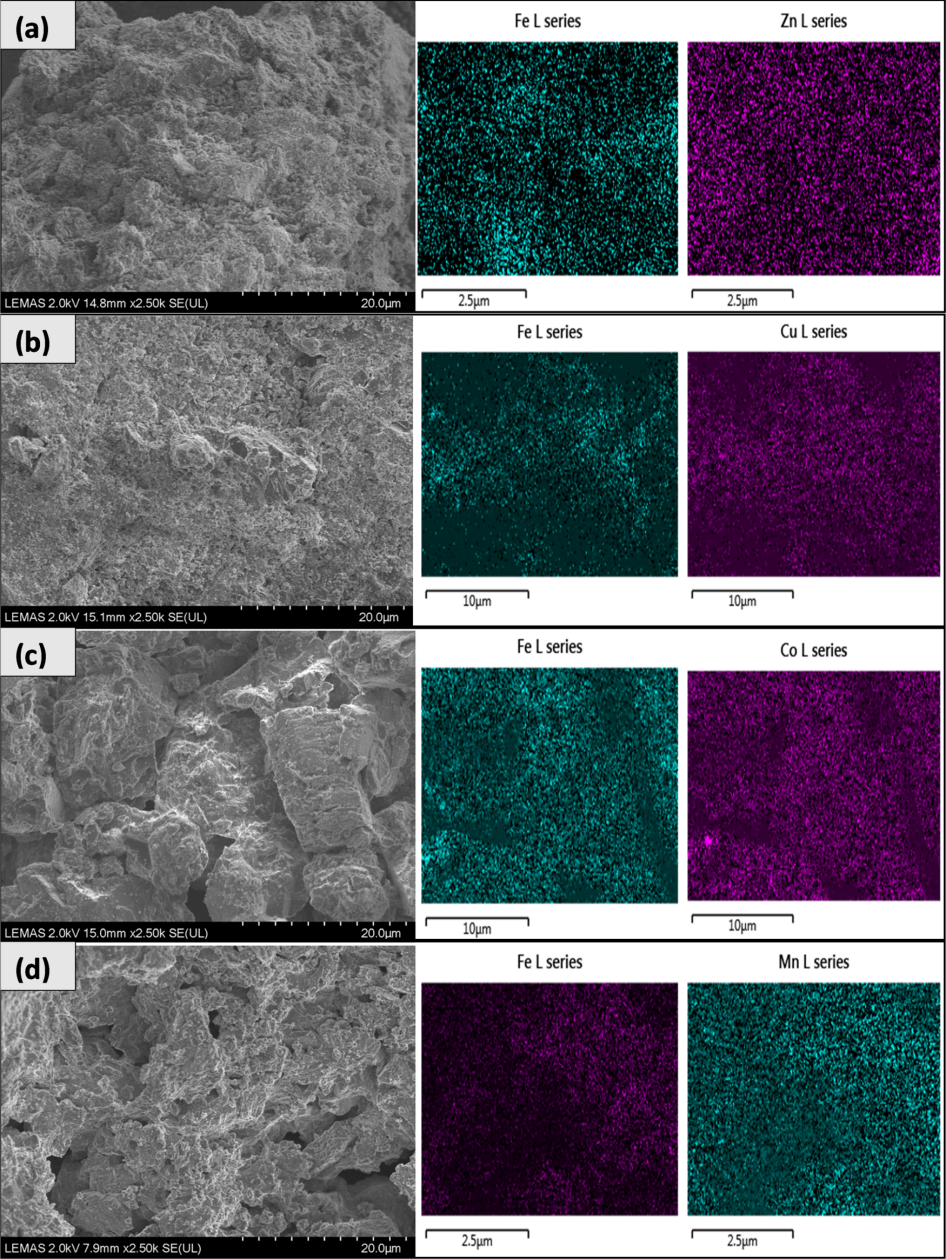
SEM–EDXS analysis of bimetallic Fe–alumina catalysts: (a) Fe–Zn/Al_2_O_3_, (b) Fe–Cu/Al_2_O_3_, (c) Fe–Co/Al_2_O_3_, and (d) Fe–Mn/Al_2_O_3_.

The properties of the bimetallic catalysts in relation to surface area and porosity are shown in Table 2. The monometallic Fe/Al_2_O_3_ catalyst had a surface area of 134 m^2^ g^–1^, and the addition of the metal (Zn, Cu, Co, Mn) to the Fe catalyst produced a moderate increase in the surface area of the bimetallic catalyst. The Cu and Co monometallic catalysts had a surface area of 89 m^2^ g^–1^ but the addition to the Fe/Al_2_O_3_ produced a marked increase in the resulting bimetallic catalyst. The pore volume of the bimetallic catalysts showed in general an increase compared to that of the monometallic catalysts. The scanning electron micrographs with accompanying EDXS metal mapping of the bimetallic-alumina catalysts are shown in Figure 8. The results show that the bimetal particles were uniformly distributed across the surface of the bimetallic catalysts for both the Fe particles and the added metal particles.

**Table 2:** Surface Area and Pore Volume of the Bimetallic Catalysts

catalyst	BET surface area (m^2^ g^–1^)	pore volume (cm^3^ g^–1^)
5 wt % Fe–5 wt % Zn/Al_2_O_3_	170	0.4141
5 wt % Fe–5 wt % Cu/Al_2_O_3_	153	0.4082
5 wt % Fe–5 wt % Co/Al_2_O_3_	153	0.4290
5 wt % Fe–5 wt % Mn/Al_2_O_3_	152	0.3631

**Figure 9:**
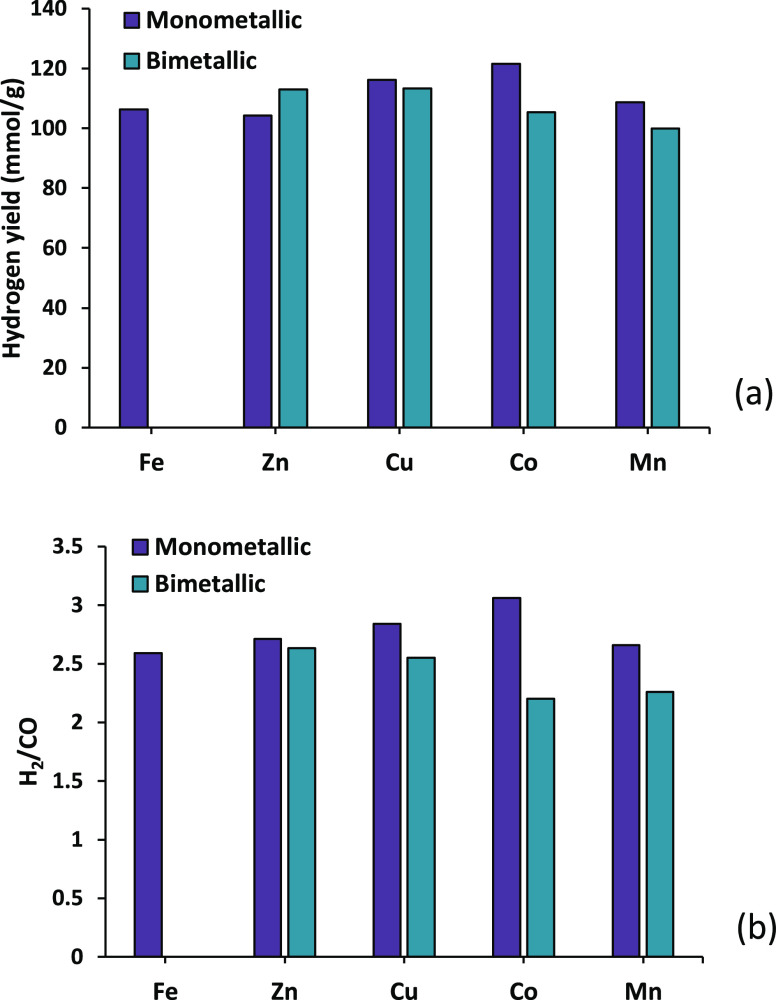
Yield of hydrogen and H_2_/CO ratio in relation to bimetallic Fe metal/Al_2_O_3_ catalysts in the third-stage (iii) water gas shift reactor.

The hydrogen yield and H_2_/CO ratio produced from the three-stage pyrolysis-catalytic steam reforming water gas shift processing of polypropylene for the bimetallic catalysts and compared with the data produced using the monometallic catalysts are shown in Figure 9. The results are presented as 10 wt % of metal loading for the monometallic catalysts and as 5 wt % of Fe plus 5 wt % of the second metal, producing 10 wt % of metal loading overall. The results show that the hydrogen yield increased for the Fe–Zn bimetallic catalyst only, with Fe–Cu showing negligible influence of the second metal and reduced hydrogen yield for the Fe–Co and Fe–Mn catalysts when compared to their monometallic equivalents (Figure 9a). There was an 8.3 and 6.3% improvement in hydrogen yield for Fe–Zn catalyst when compared to that for monometallic Zn- and Fe-catalyzed hydrogen yields, respectively. This demonstrates that the combination of Fe and Zn resulted in an interaction that ultimately increased the activity of the catalyst. Lee et al.^30^ have suggested that the addition of Zn as a catalyst promoter for the water gas shift reaction can increase the surface area and enhance the yield of hydrogen provided it is added to a suitable transition metal, particularly nickel, but less so iron. The work reported here also shows that the addition of Zn to the Fe/Al_2_O_3_ catalyst produced the highest surface area of 170 m^2^ g^–1^, which suggests that an increased surface area rather than functionality resulted in an increase in hydrogen yield. The combined Fe–Cu/Al_2_O_3_ catalyst produced a small increase in hydrogen yield from the three-stage process compared to Fe/Al_2_O_3_ alone, increasing from 106 to 113 mmol g_plastic_^–1^. However, the hydrogen yield for the Fe–Cu/Al_2_O_3_ catalyst was almost identical to that of the Cu/Al_2_O_3_ catalyst. Natesakhawat et al.^31^ also reported an increase in hydrogen yield when an iron–copper catalyst was utilized at 350 °C compared to using Fe alone. The Fe–Co/Al_2_O_3_ and Fe–Mn/Al_2_O_3_ catalysts produced lower yields of hydrogen compared to the monometallic Co/Al_2_O_3_ and Mn/Al_2_O_3_ catalysts. However, Pereira et al.^32^ reported that Co addition to Fe was an efficient promoter of the water gas shift reaction, enhancing both the activity and the textural properties of the catalyst.

Figure 9b shows the H_2_/CO ratios obtained from the three-stage processing of polypropylene in relation to the composition of the bimetallic catalysts in the third-stage (iii) water gas shift reactor. For the Fe–Zn/Al_2_O_3_ and Fe–Cu/Al_2_O_3_ catalysts, it is evident that there was no significant change in the resulting H_2_/CO ratio compared to that obtained with Fe/Al_2_O_3_ alone. Also, the H_2_/CO ratio for the Fe–Co/Al_2_O_3_ and Fe–Mn/Al_2_O_3_ catalysts produced a reduction in the H_2_/CO ratio compared to that of Fe/Al_2_O_3_ alone. This is to be expected for all bimetallic catalysts with the exception of Fe/Zn since the trends in H_2_ yield are consistent with the changes in H_2_/CO ratios. The H_2_/CO ratios for the bimetallic catalysts were in all cases lower than produced by the monometallic Zn, Cu, Co, or Mn alumina catalysts.

There was minimal influence of the addition of the promoter metals to the Fe/Al_2_O_3_ catalyst compared to the carbon deposits on the monometallic catalysts. TPO analysis of the used catalysts showed carbonaceous coke deposits to be 6.0 wt % for the Fe–Zn/Al_2_O_3_ catalyst, 6.5 wt % for the Fe–Cu/Al_2_O_3_ catalyst, 5.3 wt % for the Fe–Co/Al_2_O_3_ catalyst, and 5.7 wt % for the Fe–Mn/Al_2_O_3_ catalyst.

### 3.4 Influence of Water Gas Shift Reactor Process Conditions on Hydrogen Yield

From the previous sections, the Fe/Al_2_O_3_ catalyst has been shown to be an effective water gas shift catalyst for hydrogen production from polypropylene using the three-stage (i) pyrolysis, (ii) catalytic steam reforming, and (iii) water gas shift reactor system. In addition, Fe/Al_2_O_3_ catalysts are commonly used in commercial water gas shift reactors since they show excellent durability and cost-effectiveness. To further investigate the three-stage process, the influence of process conditions in the third-stage (iii) water gas shift reactor was investigated. The influence of the Fe metal loading on the catalyst, the input of steam flow rate, and different catalyst support materials were investigated in relation to the yield of hydrogen and the H_2_/CO ratio. The experimental conditions were a pyrolysis final temperature of 500 °C, a catalytic steam reforming temperature of 850 °C with the 10 wt % Ni/Al_2_O_3_ catalyst, and a steam input flow rate of 4 mL h^–1^. The water gas shift catalyst temperature was maintained at a higher temperature of 550 °C with a steam input flow rate of 4 mL h^–1^. The higher temperature of 550 °C was chosen as the most effective temperature for the high-temperature catalysts reported in Section 3.2.

#### 3.4.1 Influence of Catalyst Iron Loading on Hydrogen Yield

**Figure 10:**
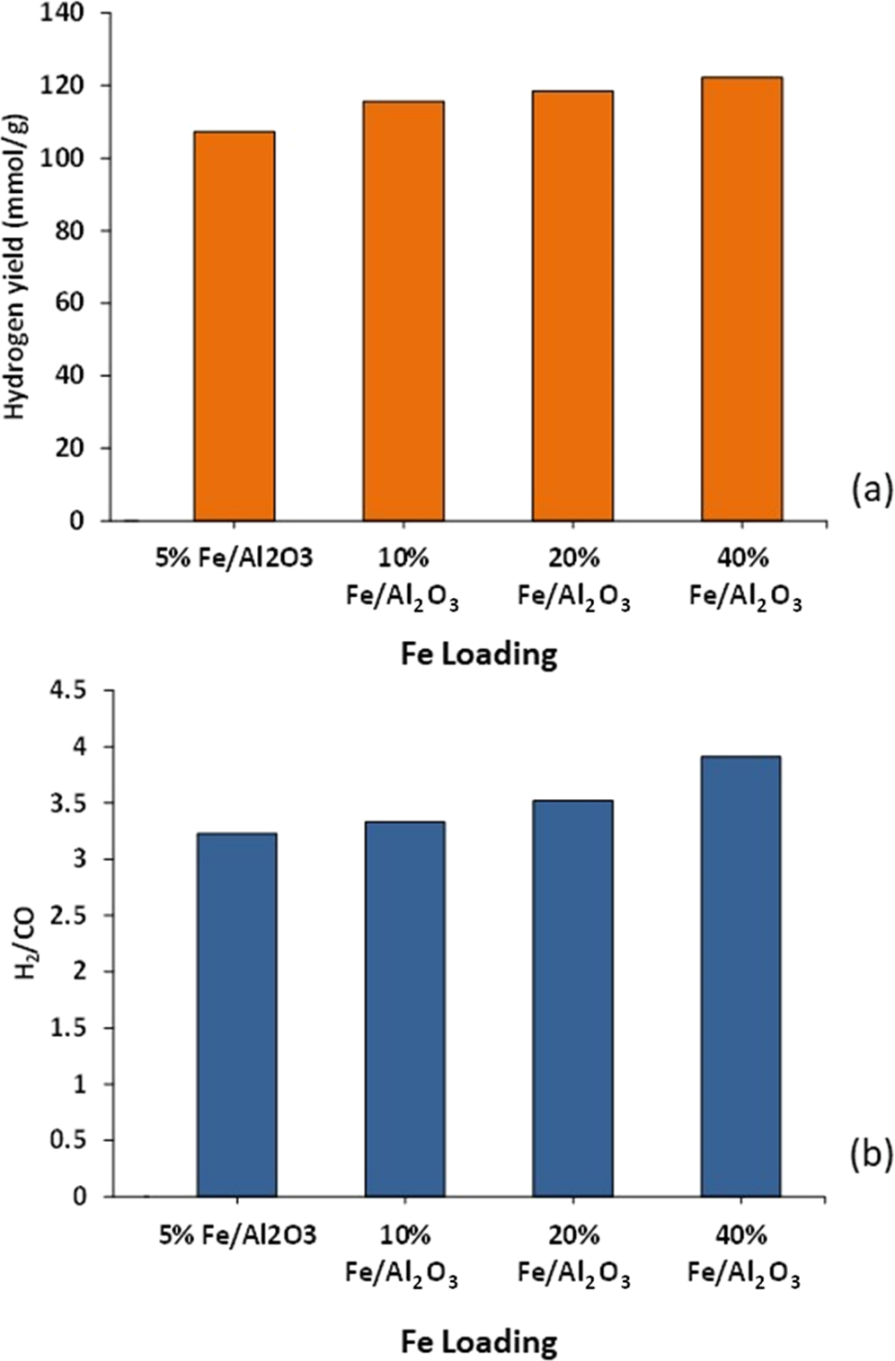
Influence of iron metal loading on the Fe/Al_2_O_3_ catalyst in the third-stage (iii) water gas shift reactor on (a) the yield of H_2_ and (b) the H_2_/CO ratio.

The influence of increasing iron loading on the Fe/Al_2_O_3_ catalyst in the third-stage (iii) water gas shift reactor was investigated using metal loadings of 5, 10, 20, and 40 wt %. Figure 10 shows the hydrogen yield and H_2_/CO ratio obtained. The results show that increasing the catalyst iron metal loading resulted in improved catalytic performance, with hydrogen yield increasing from 107 to 122 mmol g_plastic_^–1^ as the mass of iron in the catalyst was increased from 5 wt % of Fe/Al_2_O_3_ to 40 wt % of Fe/Al_2_O_3_.

The results depicted in Figure 10 show that the metal loading of 40 wt % for Fe/Al_2_O_3_ was the most favorable condition in terms of H_2_ yield; however, although the metal loading was increased from 10 to 40 wt %, the activity of the water gas shift reaction did not improve significantly. Zhu and Wachs^33^ have suggested that during the water gas shift reaction, the reactant CO chemisorption occurs on the catalyst metal sites, whereas the reactant H_2_O chemisorption occurs on the support site. As a result of increased metal loading, which increases the number of active sites that CO may chemisorb onto, it would be expected that the water gas shift reaction will be enhanced. However, the reaction becomes restricted by alumina availability when the number of active sites accessible on the active metal surface exceeds those available on the alumina surface. Therefore, increased metal loading has a lesser effect at high metal loading. Also, Figure 10 shows that the H_2_/CO ratio increased from 3.2 to 4.0 when the iron loading was increased from 5 to 40 wt %.

#### 3.4.2 Influence of Steam Flow Rate on Hydrogen Yield

**Figure 11:**
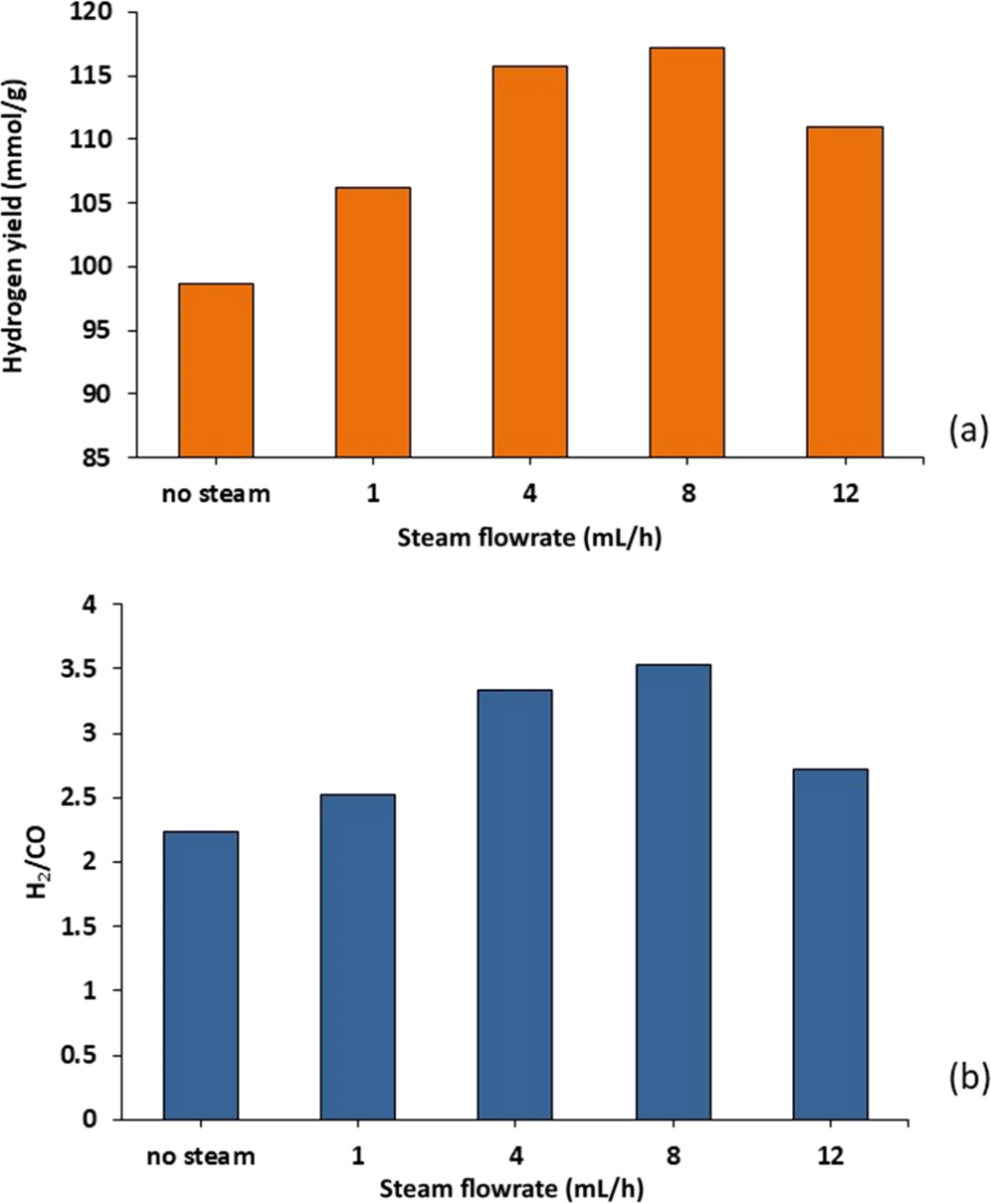
Influence of steam (water) input flow rate into the third-stage (iii) water gas shift reactor on (a) the yield of H_2_ and (b) the H_2_/CO ratio.

The influence of the input steam flow rate of 0, 1, 4, 8, and 12 mL h^–1^ on the yield of hydrogen and the H_2_/CO ratio was investigated for the three-stage (i) pyrolysis, (ii) catalytic (10 wt % Ni/Al_2_O_3_) steam reforming, and (iii) water gas shift (using the 10 wt % Fe/Al_2_O_3_ catalyst at 550 °C) process. Figure 11 shows the results obtained and shows that for no steam input, the product hydrogen yield was 98.7 mmol g_plastic_^–1^. As the steam was introduced into the third-stage (iii) water gas shift reactor, the yield of hydrogen increased to reach 117.2 mmol g_plastic_^–1^ at a steam input of 8 mL h^–1^; however, as further steam was added (12 mL h^–1^), the hydrogen yield decreased to 111.0 mmol g_plastic_^–1^. Also, the H_2_/CO ratio (Figure 11b) shows an increase with an increasing steam flow rate up to 8 mL h^–1^, but an additional increase of the steam flow rate to 12 mL h^–1^ resulted in a decrease of the H_2_/CO. When the steam injection rate was increased to 8 mL h^–1^, a significant decrease in CO and an increase in CO_2_ production occurred, suggesting that the increase in H_2_ production was driven by the enhancement of the water gas shift reaction owing to increased steam consumption. However, at higher steam inputs, the enhancement of the water gas shift reaction is inhibited by catalyst saturation as reported by several reports.^34−36^ For example, Oliveira et al.^34^ investigated the effects of changing the input steam to CO ratio on CO conversion to hydrogen in various water gas shift reactor systems using a Cu/Al_2_O_3_ catalyst. An increase in CO conversion was initially seen but higher steam input reduced CO conversion, attributed to catalyst saturation by the reactants above a critical flow rate. Park et al.^35^ and Chen et al.^36^ have also reported that a critical input of steam flow rate produces a maximum in hydrogen yield, but higher steam inputs then result in a decline in hydrogen yield.

#### 3.4.3 Influence of Catalyst Support Material on Hydrogen Loading

**Figure 12:**
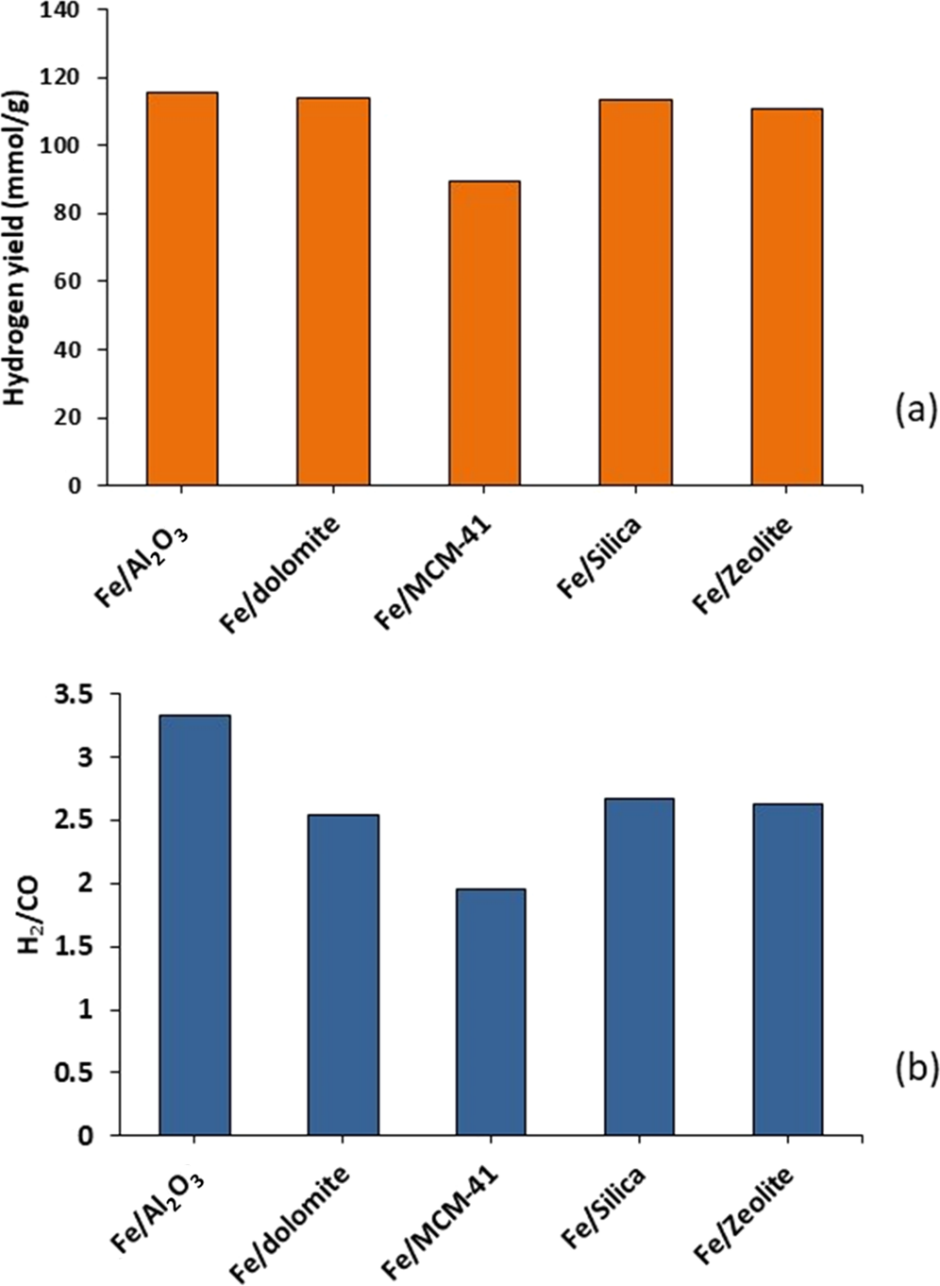
Influence of the catalytic support material used for the Fe-based catalyst used in the third-stage (iii) water gas shift reactor on (a) the yield of H_2_ and (b) the H_2_/CO ratio.

The influence of catalyst support material for the Fe-based catalyst in relation to the yield of hydrogen and the H_2_/CO ratio was investigated for the three-stage (i) pyrolysis, (ii) catalytic steam reforming, and (iii) water gas shift process. The support materials investigated for the (iii) water gas shift reaction were Al_2_O_3_, dolomite, MCM-41, silica (SiO_2_), and Y-zeolite, and the metal loading was 10 wt % of iron. The steam input to the (iii) water gas shift stage was 4 mL h^–1^, and the catalyst was maintained at a higher catalyst temperature of 550 °C. The results in terms of H_2_ yield and H_2_/CO ratio are shown in Figure 12 and show that all of the supports except MCM-41 produced relatively similar H_2_ yields of 118 mmol g_plastic_^–1^; however, the MCM-41 catalyst produced only 88 mmol g_plastic_^–1^ hydrogen yield. Figure 12b shows that Fe–Al_2_O_3_ produced the highest H_2_/CO ratio of 3.3, whereas the dolomite, silica, and Y-zeolite produced a H_2_/CO ratio of ∼2.6 and MCM-41 only a ratio of 2.0. The poor performance of MCM-41 is due to the low catalytic activity in relation to the water gas shift reaction; Tatsumi et al.^37^ and Du et al.^38^ have reported that MCM-41 is sensitive to the presence of water vapor, which promotes sintering and, therefore, deactivation. The use of Fe/SiO_2_, Fe/dolomite, and Fe/Y-zeolite as a water gas shift catalyst performed similarly to the Fe–Al_2_O_3_ catalyst in terms of hydrogen yield but has rarely been investigated in that context. Consequently, these catalysts may warrant further investigation.

Overall, the three-stage (i) pyrolysis, (ii) catalytic steam reforming, and (iii) water gas shift process for the production of hydrogen from waste plastics have shown promisingly high yields of hydrogen. There is, however, further scope for increasing the yield of hydrogen by development of the process. For example, the reactor configuration used in this work consisted of three fixed-bed reactors for each stage of the process. However, the commercial production of hydrogen from the catalytic steam reforming of natural gas methane takes place in a catalytic tubular reactor system. The use of a fluidized bed for the catalytic steam reforming reactor for the processing of plastic-derived pyrolysis hydrocarbons has produced higher yields of hydrogen at 168^22^ and 185 mmol g_plastic_^–1^.^17^ High levels of hydrogen and carbon monoxide yields from such enhanced catalytic steam reforming in the second-stage (ii) reformer would provide a high yield of carbon monoxide for the third-stage (iii) water gas shift reaction, thereby enhancing hydrogen yield via the water gas shift reaction. It should also be noted that the commercial methane reforming process takes place at significant pressures of between 0.3 and 2.5 MPa, whereas in this work, experiments were conducted at atmospheric pressure. Also in this work, the third-stage (iii) water gas shift reactor was operated at a single temperature, whereas commercially, high-temperature and separate low-temperature shift reactors are used. The operation of separate high- and low-temperature reactors for the processing of waste plastics should also enhance the overall hydrogen yield.

## 4 Conclusions

The three-stage (i) pyrolysis, (ii) catalytic steam reforming, and (iii) water gas shift processing of waste plastic for the production of hydrogen have been investigated, with emphasis on the third-stage (iii) water gas shift reaction conditions. The metal–alumina catalysts investigated in the (iii) water gas shift stage showed that Fe/Al_2_O_3_, Zn/Al_2_O_3_, and Mn/Al_2_O_3_ produced maximum hydrogen yield at the higher temperature of 550 °C, whereas the Cu/Al_2_O_3_ and Co/Al_2_O_3_ catalysts produced maximum hydrogen yield at 350 °C. Hydrogen yields at higher temperature were attributed to the promotion of reaction kinetics by the Fe/Al_2_O_3_, Zn/Al_2_O_3_, and Mn/Al_2_O_3_ catalysts, whereas at lower temperature, the exothermic nature of the water gas shift reaction resulted in a reduced CO conversion for the Cu/Al_2_O_3_ and Co/Al_2_O_3_ catalysts due to Le Chatelier’s principle dominating. Combinations of metal–alumina-based catalysts effective at high and low temperatures were investigated as bimetallic Fe–Zn, Fe–Cu, Fe–Co, and Fe–Mn catalysts. Only the Fe–Zn catalyst showed a metal–metal interaction to increase the catalytic activity of the catalyst and improve hydrogen yield.

Increasing the Fe metal loading to the water gas shift catalyst stage from 5 wt % Fe to 40 wt % Fe showed that hydrogen yield increased only by small increments, from 107 to 122 mmol g_plastic_^–1^, respectively. The results suggest that at high metal loadings, the water gas shift reaction becomes restricted by the ready availability of the active metal Fe on the alumina surface. Increasing the steam input to the third-stage water gas shift reactor (Fe/Al_2_O_2_ catalyst) produced higher hydrogen yield, together with a corresponding reduction in the CO yield and CO_2_ formation, due to the water gas shift reaction. However, at higher steam inputs, catalyst saturation resulted in a decrease in hydrogen yield. The Fe-supported catalysts, Fe/SiO_2_, Fe/dolomite, and Fe/Y-zeolite, produced very similar hydrogen yields of ∼118 mmol g_plastic_^–1^ from the three-stage processing of the plastic. However, Fe/MCM-41 showed a significantly lower activity and lower hydrogen yield, which has been attributed to the propensity of sintering of MCM-41 induced by the presence of water vapor.
